# Mol­ecular structure of tris­[(6-bromo­pyridin-2-yl)meth­yl]amine

**DOI:** 10.1107/S2056989024008685

**Published:** 2024-09-10

**Authors:** Ran Yan, Zhaohua Dai, Daniel G. Shlian, Trinit’y D. Mitchell, Aaron Loo, Kaltrina Mulosmani, Rita K. Upmacis

**Affiliations:** a Department of Chemistry Columbia University,New York New York 10027 USA; b Department of Chemistry & Physical Sciences Pace University, New York New York 10038 USA; University of Missouri-Columbia, USA

**Keywords:** crystal structure, pyrid­yl, amine, tetra­dentate tripod ligand

## Abstract

Coordination compounds of polydentate nitro­gen ligands with metals are used extensively in research areas such as catalysis, and as models of complex active sites of enzymes in bioinorganic chemistry. Tris(2-pyridyl­meth­yl)amine (TPA) is a tripodal tetra­dentate ligand that is known to form coordination compounds with metals, including copper, iron and zinc. The related compound, tris­[(6-bromo­pyridin-2-yl)meth­yl]amine (TPABr_3_), C_18_H_15_Br_3_N_4_, which possesses a bromine atom on the 6-position of each of the three pyridyl moieties, is also known but has not been heavily investigated. The mol­ecular structure of TPABr_3_ as determined by X-ray diffraction is reported here. The TPABr_3_ molecule belongs to the triclinic, *P* space group and displays interesting intermolecular Br⋯Br interactions that provide a stabilizing influence within the molecule.

## Abstract 2

Crystals of tris­[(6-bromo­pyridin-2-yl)meth­yl]amine (TPABr_3_) were obtained from a solution in aceto­nitrile upon evaporation. The crystals are triclinic with space group *P*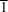.

## 1. Chemical context

Tris(2-pyridyl­meth­yl)amine (TPA) was first reported in 1967 (Anderegg & Wenk, 1967 ▸), although more recent syntheses are known (Canary *et al.*, 1998 ▸; Bazley *et al.*, 2018 ▸). TPA is a very versatile ligand and has been used to coordinate metal ions that include, for instance, copper, iron and chromium, thereby forming five- or six-coordinate complexes (Tyeklar *et al.*, 1993 ▸; Jang *et al.*, 1991 ▸; Gafford & Holwerda, 1990 ▸). A more comprehensive review of metal binding to TPA can be found elsewhere (Bravin *et al.*, 2021 ▸; Bazley *et al.*, 2018 ▸). The TPA ligand has also been successfully employed in the construction of complexes for biological models, for example, copper-cluster enzymes involved in oxygen activation (Maiti *et al.*, 2009 ▸) and in iron di­oxy­genases (Costas *et al.*, 2004 ▸). There is also an active inter­est in pursuing the synthesis of such ligands as biochemical sensors that can rapidly and selectively detect certain metals that are associated with the pathogenesis of diseases, such as Alzheimer’s disease (Jomova *et al.*, 2022 ▸; Tyczynska *et al.*, 2024 ▸). In this regard, TPA has been used to prepare piperidine compounds that can differentially chelate trace metals such as zinc and copper (Dai *et al.*, 2002 ▸). In addition to being used in biochemical sensor applications, other areas in which TPA has potential applications include anion sensors, mol­ecular switches, chiral probes and as building blocks in the synthesis of supra­molecular cages (Bravin *et al.*, 2021 ▸). Despite the prolific use of this ligand, its X-ray structure has only become available within the last ten years and has been chosen as the candidate to introduce crystallography to undergraduate students (Bats & Lerner, 2016 ▸; Bazley *et al.*, 2018 ▸).

TPA ligands containing various substituted moieties are also known. For instance, TPA containing mono-, bis-, and tris-α-methyl substitutions in the ligand complexed to FeCl_2_ have been characterized (Benhamou *et al.*, 2008 ▸). In addition, TPA ligands containing other alkyl or bromo substitutions that are also complexed to iron have been described as well as their ability to catalyze cyclo­hexane oxygenation by hydrogen peroxide (Guisado-Barrios *et al.*, 2010 ▸). Unexpectedly, a high turnover rate and efficient incorporation of oxygen from H_2_O_2_ into cyclo­hexane were reported for the iron complex of TPABr_3_, which was assumed to have the formula [Fe(TPABr_3_)(CH_3_CN)_2_]^2+^. However, a crystal structure of the TPABr_3_ ligand with or without the complexed metal has not been reported. Therefore, herein, we describe the mol­ecular structure as determined by X-ray diffraction. The synthesis of TPABr_3_ is depicted in the scheme and crystals were obtained from a solution in aceto­nitrile.
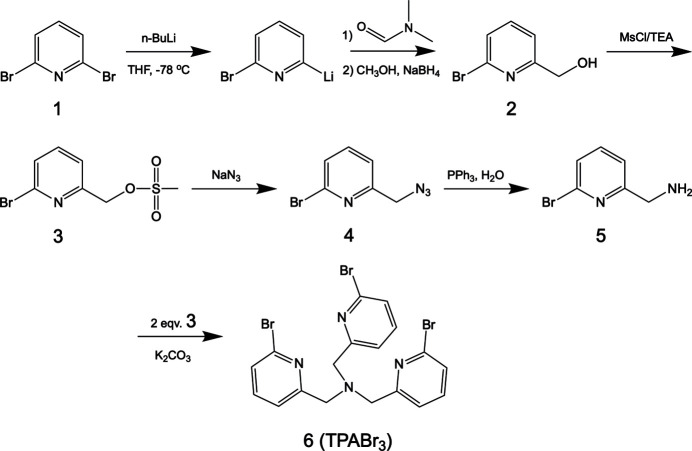


## 2. Structural commentary

**Figure 1:**
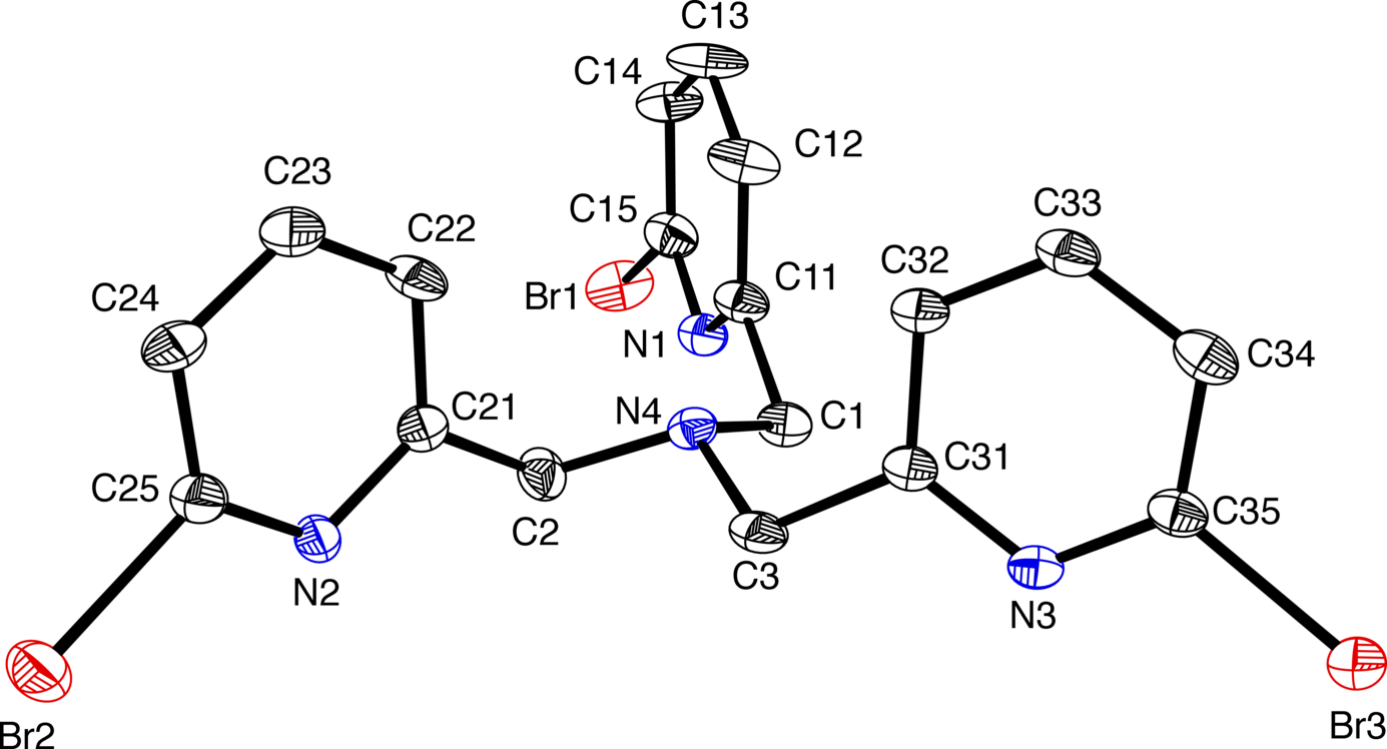
Crystal structure of TPABr_3_. Displacement ellipsoids are drawn at the 30% probability level.

The structure of TPABr_3_, shown in Fig. 1 ▸, reveals that the compound is a tertiary amine with three 6-bromo-2-methyl­pyridine subunits. The central nitro­gen atom assumes a trigonal pyramidal geometry with CH_2_—N4—CH_2_ angles ranging from 110.4 (4)–111.4 (4)°. The N4—CH_2_ distances range from 1.456 (6)–1.469 (6) Å for N4—C1, N4—C2, and N4—C3. The C—Br distances range from 1.905 (6)–1.920 (6) Å for the C15—Br1, C25—Br2 and C35—Br3 bond lengths, which compare well with the C—Br distances in the tris­(bromo­pyrazolylmeth­yl)amine ligand that measure 1.881 (5) Å (Haldón *et al.*, 2014 ▸).

## 3. Supra­molecular features

**Figure 2:**
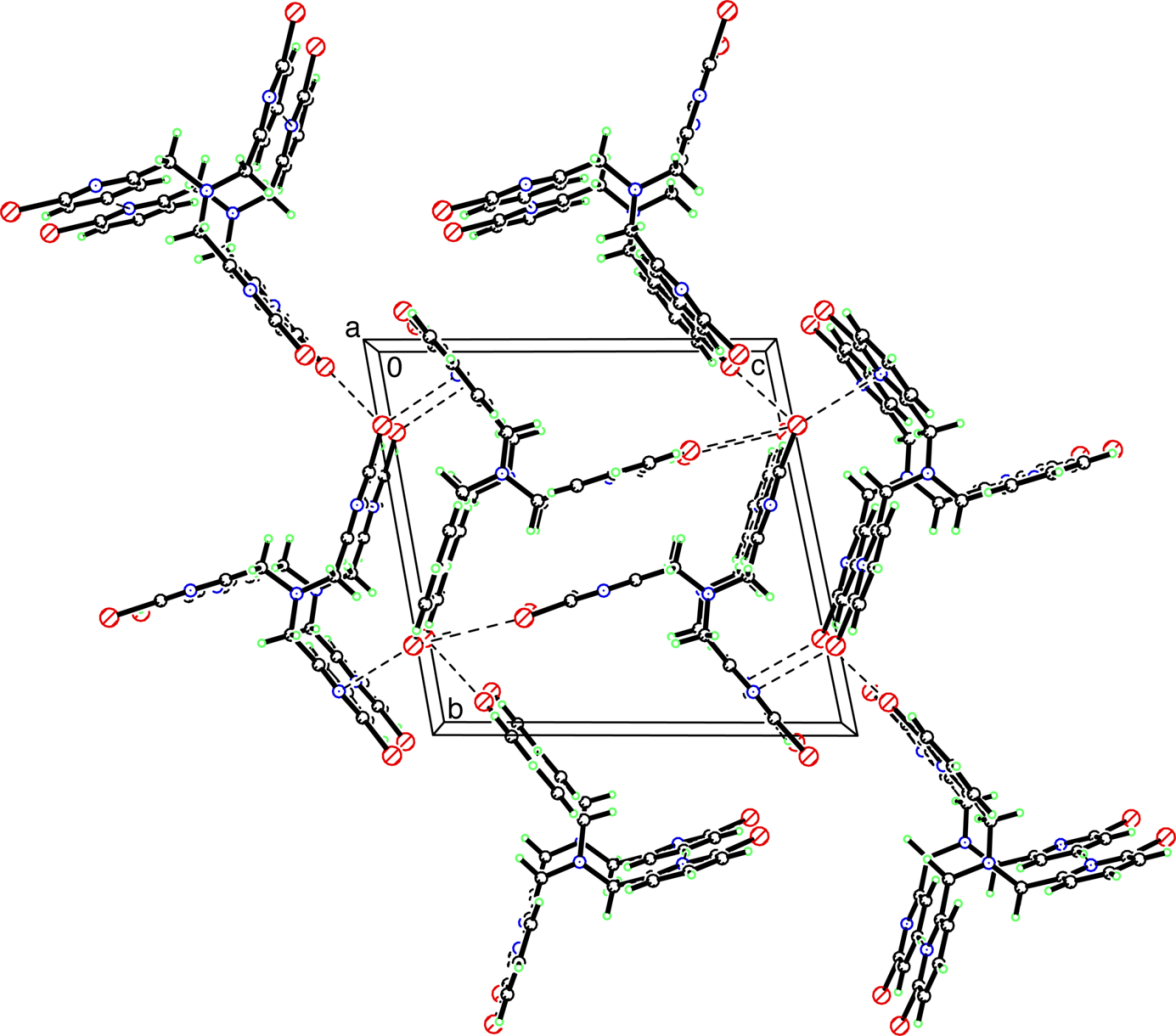
Unit-cell packing of TPABr_3_.

Fig. 2 ▸ shows the packing in the unit cell along the *a*-axis direction. There are no significant inter­molecular hydrogen-bonding inter­actions. However, there are intra­molecular distances of 2.852, 2.765 and 2.793 Å for N4⋯H12*A*, N4⋯H22*A* and N4⋯H32*A*, respectively, which at best, may indicate a very weak inter­action.

The unit cell also shows two inter­molecular Br⋯Br inter­actions, with Br2⋯Br3 at 3.6540 (11) Å and Br1⋯Br3 at 3.7731 (11) Å, which are close to the sum of the van der Waals radii, which is approximately 3.7 Å for Br⋯Br. Inter­estingly, C—Br⋯Br—C inter­actions can occur over a 3.0–4.5 Å range and provide a stabilizing influence within the crystal (Capdevila-Cortada & Novoa, 2015 ▸). The strength of the halogen–halogen inter­action depends on the halogen atom in the following order: I > Br > Cl > F (Awwadi *et al.*, 2006 ▸). It has previously been noted that *R*—Br⋯Br—*R* contacts can occur according to two different geometries, classified as type I (symmetrical inter­actions where θ_1_ = θ_2_) and type II (bent inter­actions where θ_1_ ≃180° and θ_2_ ≃90°) (Sakurai *et al.*, 1963 ▸; Desiraju & Parthasarathy, 1989 ▸; Cavallo *et al.*, 2016 ▸). In TPABr_3_, θ_1_ and θ_2_ are 160.82 and 74.14° for C15—Br1⋯Br3—C35, and 176.97 and 87.15° for C25—Br2⋯Br3—C35, respectively, indicating that they are type II inter­actions.

For comparison, the packing in the unit cell of the related tris­(bromopyrazolylmeth­yl)amine ligand is arranged in a different fashion, displaying inter­molecular pyrazolyl N⋯Br distances of 3.099 Å (Haldón *et al.*, 2014 ▸). The N—C—N—N torsion angle (from the central nitro­gen atom to the nitro­gen atoms in the pyrazolyl ring) is between 95.20 and 95.25° compared to the corresponding values of 122.2 (5)–132.6 (5)° in TPABr_3_ for the N—C—C—N bonds, indicating the different degrees of rotation of the pyrazolyl *versus* pyridyl rings.

## 4. Database survey

Much effort has been expended synthesizing TPA ligands that contain novel substitutions on the pyridyl rings. For instance, TPA derivatives containing the following types of groups have been reported: (i) tripodal tetra­dentate ligands containing pyridyl-pivalamido groups have been prepared and complexed to copper and zinc ions (Harata *et al.*, 1998 ▸; Rivas *et al.*, 2003 ▸); (ii) TPA ligands containing pyridyl-tri­meth­oxy­phenyl groups have been synthesized (and complexed with copper and zinc ions) in an effort to enhance their solubility in aqueous and common organic solvents (Liang *et al.*, 2009 ▸); (iii) TPA-related derivatives containing carb­oxy­lic acid functionalities on the pyridyl rings have been synthesized and their complexation to gadolinium investigated (Bretonnière *et al.*, 2001 ▸); (iv) a TPA derivative containing thio­urea substitutions has been prepared and coordinated with different transition metal ions, forming seven co-ordinate Mn^II^ and Cd^II^, six co-ordinate Ni^II^ and five co-ordinate Co^II^, Cu^II^ and Zn^II^ complexes (Saad *et al.*, 2012 ▸); (v) sulfonyl subunits have been attached to the pyridyl rings in order to make TPA highly water compatible, which allows for broader applicability to the biomedical arena (Salaam *et al.*, 2020 ▸); (vi) iso­quinoline-derivatized TPAs have been prepared for use as fluorescent zinc sensors (Mikata *et al.*, 2014 ▸, 2015 ▸), and (vii) other TPA-based ligands that have been prepared include those possessing phenyl­ethynyl units and their copper(II) complexes investigated (Lim *et al.*, 2016 ▸). Furthermore, TPA ligands containing one and two chiral substituents on the tripodal skeleton have been synthesized using lipase enzyme and lanthanide complexation investigated (Yamada *et al.*, 2003 ▸).

Tripod ligands containing pyrazolyl rather than pyridyl rings are also known. In this regard, novel tris­(pyrazolylmeth­yl)amine ligands that contain methyl and bromo substituents on the pyrazolyl moiety have been synthesized and structurally characterized (Haldón *et al.*, 2014 ▸). The catalytic activities of the copper(I) complexes of these ligands were explored in carbene- and nitrene-transfer studies. In this case, the crystal structure for the tris­(bromo-pyrazolylmeth­yl)amine ligand is known (Haldón *et al.*, 2014 ▸).

## 5. Synthesis and crystallization

The synthesis of TPABr_3_ is shown in the Scheme. The starting material, 2,6-di­bromo­pyridine (Compound **1**), was reacted with *n*-butyl­lithium at 195 K to generate 2-bromo-6-li­thio­pyridine (not isolated), which was subsequently reacted with DMF followed by reduction with NaBH_4_ to give (6-bromo-2-pyrid­yl)methanol (compound **2**). Using methane­sulfonyl chloride (MsCl) and tri­ethyl­amine (TEA), the alcohol compound **2** was converted to a mesylate compound **3**. Mesylate **3** was reacted with NaN_3_ in an S_N_2 reaction to afford the organic azide (compound **4**), which was subsequently reduced by PPh_3_ to a primary amine (compound **5**). Reacting compound **5** with two equivalents of mesylate compound **3** resulted in the target compound TPABr_3_**6**, with an overall yield of 49%.

The resulting compound, TPABr_3_ (0.0133 g; 0.024 mmol), was dissolved in aceto­nitrile (CH_3_CN; 2 mL) and allowed to evaporate. After 4 days at ambient temperature, colorless needles of TPABr_3_, suitable for X-ray diffraction, were crystallized from the solution.

## 6. Refinement

Crystal data, data collection and structure refinement details are summarized in Table 1 ▸. Hydrogen atoms on carbon were placed in calculated positions and included as riding contributions with isotropic displacement parameters *U*_iso_(H) = 1.2*U*_eq_(C*sp*^2^) or 1.5*U*_eq_(C*sp*^3^) using *SHELXL2019/1* (Sheldrick, 2015*b* ▸). The structure contained poorly defined aceto­nitrile solvent mol­ecules that were removed by the SQUEEZE procedure in *PLATON* (Spek, 2015 ▸), which identified a void volume of 167 Å^3^ containing approximately 47 electrons.

## Supplementary Material

Crystal structure: contains datablock(s) I. DOI: 10.1107/S2056989024008685/ev2008sup1.cif

Structure factors: contains datablock(s) I. DOI: 10.1107/S2056989024008685/ev2008Isup4.hkl

Supporting information file. DOI: 10.1107/S2056989024008685/ev2008Isup3.cml

CCDC reference: 2381692

Additional supporting information:  crystallographic information; 3D view; checkCIF report

## Figures and Tables

**Table 1:** Experimental details

Crystal data	
Chemical formula	C_18_H_15_Br_3_N_4_
*M*_r_	527.07
Crystal system, space group	Triclinic, *P* 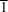
Temperature (K)	180
*a*, *b*, *c* (Å)	6.2445 (8), 13.2335 (16), 13.4984 (16)
α, β, γ (°)	79.168 (2), 88.671 (2), 78.962 (2)
*V* (Å^3^)	1075.2 (2)
*Z*	2
Radiation type	Mo *K*α
μ (mm^−1^)	5.64
Crystal size (mm)	0.15 × 0.13 × 0.04

Data collection	
Diffractometer	Bruker APEXII CCD
Absorption correction	Multi-scan (*SADABS*; Krause *et al.*, 2015 ▸)
*T*_min_, *T*_max_	0.535, 0.746
No. of measured, independent and observed [*I* > 2σ(*I*)] reflections	11638, 3813, 2434
*R*_int_	0.070
(sin θ/λ)_max_ (Å^−1^)	0.595

Refinement	
*R*[*F*^2^ > 2σ(*F*^2^)], *wR*(*F*^2^), *S*	0.045, 0.114, 1.02
No. of reflections	3813
No. of parameters	226
H-atom treatment	H-atom parameters constrained
Δρ_max_, Δρ_min_ (e Å^−3^)	0.47, −0.67

Computer programs: *APEX4* and *SAINT* (Bruker, 2014 ▸), *SHELXT* (Sheldrick 2015*a* ▸), *SHELXL2019/1* (Sheldrick, 2015*b* ▸) and *SHELXTL* (Sheldrick, 2008 ▸).

## supplementary crystallographic information

### Tris[(6-bromopyridin-2-yl)methyl]amine Crystal data

**Table:**

C_18_H_15_Br_3_N_4_	*Z* = 2
*M**_r_* = 527.07	*F*(000) = 512
Triclinic, *P*1	*D*_x_ = 1.628 Mg m^−^^3^
*a* = 6.2445 (8) Å	Mo *K*α radiation, λ = 0.71073 Å
*b* = 13.2335 (16) Å	Cell parameters from 3031 reflections
*c* = 13.4984 (16) Å	θ = 3.1–23.8°
α = 79.168 (2)°	µ = 5.64 mm^−^^1^
β = 88.671 (2)°	*T* = 180 K
γ = 78.962 (2)°	Plate, colorless
*V* = 1075.2 (2) Å^3^	0.15 × 0.13 × 0.04 mm

### Tris[(6-bromopyridin-2-yl)methyl]amine Data collection

**Table:**

Bruker APEXII CCD diffractometer	2434 reflections with *I* > 2σ(*I*)
φ and ω scans	*R*_int_ = 0.070
Absorption correction: multi-scan (SADABS; Krause *et al.*, 2015)	θ_max_ = 25.0°, θ_min_ = 1.5°
*T*_min_ = 0.535, *T*_max_ = 0.746	*h* = −7→7
11638 measured reflections	*k* = −15→15
3813 independent reflections	*l* = −16→16

### Tris[(6-bromopyridin-2-yl)methyl]amine Refinement

**Table:**

Refinement on *F*^2^	Primary atom site location: dual
Least-squares matrix: full	Hydrogen site location: inferred from neighbouring sites
*R*[*F*^2^ > 2σ(*F*^2^)] = 0.045	H-atom parameters constrained
*wR*(*F*^2^) = 0.114	*w* = 1/[σ^2^(*F*_o_^2^) + (0.0464*P*)^2^] where *P* = (*F*_o_^2^ + 2*F*_c_^2^)/3
*S* = 1.02	(Δ/σ)_max_ < 0.001
3813 reflections	Δρ_max_ = 0.47 e Å^−^^3^
226 parameters	Δρ_min_ = −0.67 e Å^−^^3^
0 restraints	

### Tris[(6-bromopyridin-2-yl)methyl]amine Special details

**Table:**

Geometry. All esds (except the esd in the dihedral angle between two l.s. planes) are estimated using the full covariance matrix. The cell esds are taken into account individually in the estimation of esds in distances, angles and torsion angles; correlations between esds in cell parameters are only used when they are defined by crystal symmetry. An approximate (isotropic) treatment of cell esds is used for estimating esds involving l.s. planes.

### Tris[(6-bromopyridin-2-yl)methyl]amine Fractional atomic coordinates and isotropic or equivalent isotropic displacement parameters (Å^2^)

**Table:**

	*x*	*y*	*z*	*U*_iso_*/*U*_eq_	
N1	0.2019 (7)	0.3566 (4)	0.5337 (3)	0.0361 (12)	
N2	0.2962 (7)	0.0992 (3)	0.2191 (3)	0.0326 (11)	
N3	0.1478 (7)	0.5757 (4)	0.0911 (3)	0.0297 (11)	
N4	0.2814 (7)	0.3485 (3)	0.2805 (3)	0.0289 (11)	
C1	0.1888 (9)	0.4160 (5)	0.3517 (4)	0.0339 (14)	
H1B	0.033813	0.410977	0.363630	0.041*	
H1C	0.195167	0.489831	0.322584	0.041*	
C2	0.2338 (9)	0.2435 (4)	0.3100 (4)	0.0375 (14)	
H2B	0.082095	0.244305	0.289608	0.045*	
H2C	0.245189	0.222326	0.384317	0.045*	
C3	0.2015 (8)	0.3948 (4)	0.1774 (4)	0.0352 (14)	
H3B	0.040155	0.413243	0.177665	0.042*	
H3C	0.242935	0.342154	0.133650	0.042*	
C11	0.3149 (9)	0.3831 (5)	0.4508 (4)	0.0362 (14)	
C12	0.5393 (10)	0.3798 (5)	0.4535 (5)	0.0520 (18)	
H12A	0.615838	0.397766	0.393057	0.062*	
C13	0.6481 (10)	0.3503 (6)	0.5449 (5)	0.062 (2)	
H13A	0.800619	0.348382	0.548534	0.074*	
C14	0.5330 (9)	0.3234 (5)	0.6310 (5)	0.0505 (18)	
H14A	0.602337	0.302168	0.695444	0.061*	
C15	0.3143 (10)	0.3290 (5)	0.6190 (4)	0.0426 (15)	
C21	0.3881 (8)	0.1643 (4)	0.2622 (4)	0.0309 (13)	
C22	0.6111 (9)	0.1585 (5)	0.2635 (4)	0.0398 (15)	
H22A	0.671672	0.206481	0.293351	0.048*	
C23	0.7461 (9)	0.0826 (5)	0.2212 (4)	0.0446 (16)	
H23A	0.899873	0.077695	0.221881	0.054*	
C24	0.6541 (9)	0.0143 (5)	0.1780 (5)	0.0444 (16)	
H24A	0.740674	−0.039675	0.149128	0.053*	
C25	0.4311 (9)	0.0285 (5)	0.1790 (5)	0.0407 (15)	
C31	0.2914 (8)	0.4916 (4)	0.1340 (4)	0.0275 (13)	
C32	0.5142 (8)	0.4917 (5)	0.1382 (4)	0.0339 (14)	
H32A	0.612525	0.430920	0.170020	0.041*	
C33	0.5894 (9)	0.5802 (4)	0.0960 (4)	0.0355 (14)	
H33A	0.740996	0.581301	0.097381	0.043*	
C34	0.4421 (9)	0.6686 (5)	0.0512 (4)	0.0367 (15)	
H34A	0.488557	0.731787	0.021853	0.044*	
C35	0.2253 (8)	0.6605 (5)	0.0512 (4)	0.0322 (14)	
Br1	0.14783 (11)	0.28852 (6)	0.73619 (5)	0.0588 (2)	
Br2	0.29223 (11)	−0.06206 (6)	0.11734 (6)	0.0598 (2)	
Br3	0.01178 (10)	0.77792 (5)	−0.00858 (5)	0.0454 (2)	

### Tris[(6-bromopyridin-2-yl)methyl]amine Atomic displacement parameters (Å^2^)

**Table:**

	*U*^11^	*U*^22^	*U*^33^	*U*^12^	*U*^13^	*U*^23^
N1	0.031 (3)	0.042 (3)	0.038 (3)	−0.008 (2)	0.001 (2)	−0.013 (2)
N2	0.024 (2)	0.029 (3)	0.048 (3)	−0.006 (2)	0.002 (2)	−0.013 (2)
N3	0.027 (3)	0.037 (3)	0.026 (2)	−0.008 (2)	−0.003 (2)	−0.007 (2)
N4	0.026 (2)	0.034 (3)	0.027 (3)	−0.003 (2)	−0.0025 (19)	−0.009 (2)
C1	0.032 (3)	0.046 (4)	0.026 (3)	−0.010 (3)	0.000 (2)	−0.008 (3)
C2	0.035 (3)	0.034 (4)	0.046 (4)	−0.012 (3)	0.008 (3)	−0.007 (3)
C3	0.021 (3)	0.046 (4)	0.042 (4)	−0.011 (3)	0.000 (2)	−0.012 (3)
C11	0.027 (3)	0.047 (4)	0.039 (4)	−0.010 (3)	0.004 (3)	−0.020 (3)
C12	0.037 (4)	0.081 (5)	0.047 (4)	−0.026 (4)	0.004 (3)	−0.020 (4)
C13	0.022 (3)	0.109 (6)	0.062 (5)	−0.024 (4)	−0.002 (3)	−0.022 (4)
C14	0.032 (4)	0.078 (5)	0.044 (4)	−0.007 (3)	−0.008 (3)	−0.017 (4)
C15	0.039 (4)	0.051 (4)	0.042 (4)	−0.014 (3)	0.002 (3)	−0.017 (3)
C21	0.029 (3)	0.033 (3)	0.030 (3)	−0.004 (3)	0.001 (2)	−0.005 (3)
C22	0.029 (3)	0.050 (4)	0.048 (4)	−0.021 (3)	0.002 (3)	−0.012 (3)
C23	0.023 (3)	0.050 (4)	0.061 (4)	−0.008 (3)	0.004 (3)	−0.010 (4)
C24	0.020 (3)	0.049 (4)	0.064 (4)	−0.001 (3)	0.003 (3)	−0.017 (3)
C25	0.033 (3)	0.037 (4)	0.052 (4)	−0.010 (3)	−0.007 (3)	−0.003 (3)
C31	0.028 (3)	0.037 (4)	0.019 (3)	−0.009 (3)	0.001 (2)	−0.009 (3)
C32	0.022 (3)	0.042 (4)	0.038 (3)	−0.007 (3)	0.000 (2)	−0.008 (3)
C33	0.024 (3)	0.044 (4)	0.044 (4)	−0.011 (3)	0.003 (3)	−0.017 (3)
C34	0.033 (3)	0.047 (4)	0.037 (3)	−0.018 (3)	0.009 (3)	−0.017 (3)
C35	0.029 (3)	0.040 (4)	0.033 (3)	−0.010 (3)	0.001 (2)	−0.016 (3)
Br1	0.0457 (4)	0.0890 (6)	0.0383 (4)	−0.0084 (4)	0.0041 (3)	−0.0086 (4)
Br2	0.0428 (4)	0.0583 (5)	0.0889 (6)	−0.0087 (3)	−0.0046 (4)	−0.0410 (4)
Br3	0.0343 (4)	0.0423 (4)	0.0574 (4)	−0.0095 (3)	−0.0085 (3)	−0.0009 (3)

### Tris[(6-bromopyridin-2-yl)methyl]amine Geometric parameters (Å, º)

**Table:**

N1—C15	1.317 (7)	C13—C14	1.378 (8)
N1—C11	1.335 (7)	C14—C15	1.366 (8)
N2—C25	1.320 (7)	C15—Br1	1.920 (6)
N2—C21	1.343 (6)	C21—C22	1.381 (7)
N3—C35	1.324 (6)	C22—C23	1.384 (8)
N3—C31	1.334 (6)	C23—C24	1.378 (8)
N4—C2	1.456 (6)	C24—C25	1.369 (7)
N4—C1	1.468 (6)	C25—Br2	1.920 (6)
N4—C3	1.469 (6)	C31—C32	1.394 (7)
C1—C11	1.516 (7)	C32—C33	1.365 (7)
C2—C21	1.511 (7)	C33—C34	1.389 (8)
C3—C31	1.509 (7)	C34—C35	1.378 (7)
C11—C12	1.395 (8)	C35—Br3	1.905 (6)
C12—C13	1.376 (8)		
			
C15—N1—C11	116.0 (5)	N2—C21—C22	121.9 (5)
C25—N2—C21	116.3 (5)	N2—C21—C2	116.2 (5)
C35—N3—C31	117.2 (4)	C22—C21—C2	121.9 (5)
C2—N4—C1	111.4 (4)	C21—C22—C23	119.7 (5)
C2—N4—C3	110.4 (4)	C24—C23—C22	119.0 (5)
C1—N4—C3	110.9 (4)	C25—C24—C23	116.4 (6)
N4—C1—C11	110.2 (4)	N2—C25—C24	126.7 (5)
N4—C2—C21	111.9 (4)	N2—C25—Br2	114.8 (4)
N4—C3—C31	112.6 (4)	C24—C25—Br2	118.5 (5)
N1—C11—C12	122.4 (5)	N3—C31—C32	122.2 (5)
N1—C11—C1	117.0 (5)	N3—C31—C3	116.7 (4)
C12—C11—C1	120.6 (5)	C32—C31—C3	121.1 (5)
C13—C12—C11	119.0 (6)	C33—C32—C31	119.3 (5)
C12—C13—C14	119.1 (6)	C32—C33—C34	119.3 (5)
C15—C14—C13	116.6 (6)	C35—C34—C33	116.9 (5)
N1—C15—C14	126.8 (6)	N3—C35—C34	125.1 (5)
N1—C15—Br1	114.9 (4)	N3—C35—Br3	115.1 (4)
C14—C15—Br1	118.3 (5)	C34—C35—Br3	119.8 (4)
			
C2—N4—C1—C11	71.5 (5)	N4—C2—C21—C22	47.9 (7)
C3—N4—C1—C11	−165.2 (4)	N2—C21—C22—C23	−1.3 (9)
C1—N4—C2—C21	−159.9 (4)	C2—C21—C22—C23	178.2 (5)
C3—N4—C2—C21	76.5 (5)	C21—C22—C23—C24	0.3 (9)
C2—N4—C3—C31	−166.5 (4)	C22—C23—C24—C25	1.1 (9)
C1—N4—C3—C31	69.6 (5)	C21—N2—C25—C24	0.7 (9)
C15—N1—C11—C12	1.3 (9)	C21—N2—C25—Br2	−179.5 (4)
C15—N1—C11—C1	−179.6 (5)	C23—C24—C25—N2	−1.7 (10)
N4—C1—C11—N1	−122.2 (5)	C23—C24—C25—Br2	178.6 (5)
N4—C1—C11—C12	57.0 (7)	C35—N3—C31—C32	0.6 (7)
N1—C11—C12—C13	−1.3 (10)	C35—N3—C31—C3	−178.9 (4)
C1—C11—C12—C13	179.6 (6)	N4—C3—C31—N3	−131.2 (5)
C11—C12—C13—C14	0.8 (11)	N4—C3—C31—C32	49.3 (7)
C12—C13—C14—C15	−0.4 (10)	N3—C31—C32—C33	−0.9 (8)
C11—N1—C15—C14	−0.9 (9)	C3—C31—C32—C33	178.6 (5)
C11—N1—C15—Br1	−178.7 (4)	C31—C32—C33—C34	0.9 (8)
C13—C14—C15—N1	0.5 (10)	C32—C33—C34—C35	−0.7 (8)
C13—C14—C15—Br1	178.2 (5)	C31—N3—C35—C34	−0.4 (8)
C25—N2—C21—C22	0.8 (8)	C31—N3—C35—Br3	−179.9 (4)
C25—N2—C21—C2	−178.7 (5)	C33—C34—C35—N3	0.5 (8)
N4—C2—C21—N2	−132.6 (5)	C33—C34—C35—Br3	180.0 (4)
